# Preventing a cluster from becoming a new wave in settings with zero community COVID-19 cases

**DOI:** 10.1186/s12879-022-07180-1

**Published:** 2022-03-07

**Authors:** Romesh G. Abeysuriya, Dominic Delport, Robyn M. Stuart, Rachel Sacks-Davis, Cliff C. Kerr, Dina Mistry, Daniel J. Klein, Margaret Hellard, Nick Scott

**Affiliations:** 1 Burnet Institute, Melbourne, VIC Australia; 2 Department of Epidemiology and Preventive Medicine, Monash University, Melbourne, Australia; 3 Department of Mathematical Sciences, University of Copenhagen, Copenhagen, Denmark; 4 Institute for Disease Modeling at the Bill & Melinda Gates Foundation, Seattle, USA; 5 School of Physics, University of Sydney, Sydney, NSW Australia

**Keywords:** COVID-19, Modeling, Outbreaks, Containment strategy

## Abstract

**Background:**

In settings with zero community transmission, any new SARS-CoV-2 outbreaks are likely to be the result of random incursions. The level of restrictions in place at the time of the incursion is likely to considerably affect possible outbreak trajectories, but the probability that a large outbreak eventuates is not known.

**Methods:**

We used an agent-based model to investigate the relationship between ongoing restrictions and behavioural factors, and the probability of an incursion causing an outbreak and the resulting growth rate. We applied our model to the state of Victoria, Australia, which has reached zero community transmission as of November 2020.

**Results:**

We found that a future incursion has a 45% probability of causing an outbreak (defined as a 7-day average of > 5 new cases per day within 60 days) if no restrictions were in place, decreasing to 23% with a mandatory masks policy, density restrictions on venues such as restaurants, and if employees worked from home where possible. A drop in community symptomatic testing rates was associated with up to a 10-percentage point increase in outbreak probability, highlighting the importance of maintaining high testing rates as part of a suppression strategy.

**Conclusions:**

Because the chance of an incursion occurring is closely related to border controls, outbreak risk management strategies require an integrated approaching spanning border controls, ongoing restrictions, and plans for response. Each individual restriction or control strategy reduces the risk of an outbreak. They can be traded off against each other, but if too many are removed there is a danger of accumulating an unsafe level of risk. The outbreak probabilities estimated in this study are of particular relevance in assessing the downstream risks associated with increased international travel.

**Supplementary Information:**

The online version contains supplementary material available at 10.1186/s12879-022-07180-1.

## Background

Coronavirus disease 2019 (COVID-19) has had dramatic social and economic impacts since the WHO declared it a pandemic in March 2020 [1]. Caused by the severe acute respiratory syndrome Coronavirus-2 (SARS-CoV-2) virus, COVID-19 is particularly difficult to contain because of its high reproduction number in the range of 2.25–3.5 [2, 3], variable and potentially long incubation period [4–6], and because of pre-symptomatic/asymptomatic transmission [7, 8]. Several effective vaccines were developed in late 2020 [9–12] but with limited availability and logistical challenges in global distribution [13], in the near future non-pharmaceutical interventions will continue to remain the primary mechanism for managing the pandemic in many countries.

Strategies to contain SARS-CoV-2 vary from setting to setting, depending on epidemiological factors, health system capacity, political will, and the economic feasibility of introducing or maintaining physical distancing restrictions. In settings with high prevalence, policy decisions are heavily guided by epidemic indicators such as the number of diagnoses per day, or the number of people hospitalized and the health system capacity. Policy changes affect these indicators within weeks, so these measures provide feedback to guide scaling interventions up or down. However, in settings such as New Zealand and Taiwan, that have effectively eliminated community transmission of the virus as of December 2020, the situation is different. With zero community transmission, epidemic indicators are not available to guide policy, and the risk to the community is difficult to quantify since even large, high-mixing events will not lead to an outbreak. Nonetheless, a level of risk persists due to the possibility that an external source can seed an undiagnosed infection and lead to an outbreak. This may have important consequences if the outbreak grows too rapidly or is detected too late to be contained with a combination of testing, contact tracing and quarantine. Such an outbreak would likely occur without any advance warning– for example, an individual who has returned from overseas and was quarantined, but remained undetected but infectious after the prescribed quarantine period (usually 14 days), or a failure in quarantine procedure leading to a transmission event to support staff during quarantine. These kinds of events have now been observed on several occasions in low prevalence settings, including recent Australian outbreaks in Melbourne, Adelaide, and Sydney, with the Melbourne outbreak leading to considerable restrictions on movement and social mixing that lasted almost 6 months.

If an undetected infection is introduced to a setting with no community transmission, three broad outcomes are possible: no/limited transmission occurs, and the outbreak dissipates naturally (perhaps even without being diagnosed and detected); transmission occurs leading to a cluster of infections that is eventually contained with testing, contact tracing and quarantine; or sufficient transmission occurs to create sustained epidemic growth such that additional restrictions or interventions are required to regain epidemic control. Which outcome will occur is stochastic, and depends on a number of factors including the extent of restrictions imposed at the time (and hence the extent of individual-level mixing that is allowed), the capacity and efficiency of the testing and contact tracing system, compliance with quarantine directions, any non-pharmaceutical interventions in place at the time (e.g. physical distancing policies, mask coverage), and the socio-demographic and contact networks of the first few infected individuals.

A range of outbreak outcomes have been seen in various settings over the second half of 2020. In Australia, following an initial wave of SARS-CoV-2 infections in March–April 2020, a variety of restrictions and public health measures were imposed to reduce transmission, and by May 2020 all Australian jurisdictions had negligible community transmission. After easing restrictions, in late June the state of Victoria experienced an epidemic resurgence requiring restrictions to be re-imposed between July and August, with a second wave peak of 687 diagnoses on 4th August [14]. Almost all cases were able to be traced back to just four incursion events. Over the same period, the states of NSW and Queensland also detected instances of community transmission. However, in these states the outbreak was controlled or contained with testing and contact tracing, without requiring major restrictions to be imposed. Similarly, New Zealand experienced several incursion events after reaching zero community transmission. In August 2020, a single infection triggered a three-week lockdown in Auckland, with 179 downstream infections in total [15], but subsequent incursions in October and November did not spread widely and were contained without community restrictions. These different examples highlight how a range of different outcomes are possible when new cases are seeded, even under similar circumstances.

Following the late-2020 outbreak in Victoria, the state has now achieved zero detected community transmission [14]. As it moves into this transmission regime, decisions need to be made on which restrictions or interventions should be maintained longer-term to balance the competing needs of minimizing outbreak risk and maximizing social and economic freedoms. Maintaining heavier restrictions is likely to significantly reduce the risk of an outbreak if a new case is introduced but would have unsustainable social and economic costs. On the other hand, with no restrictions in place, high levels of mixing and possible relaxed behaviours combined with likely low testing rates due to reduced perceived likelihood of virus transmission, it is possible that a newly introduced case could go unnoticed for weeks, and hence that the introduction of a single case could lead to an uncontrolled outbreak. A happy medium may be a set of lighter restrictions (e.g. limits on large events, mandatory masks in places such as public transport and enclosed public spaces) that have a lower social and economic cost but could still reduce outbreak risk in a meaningful way. To inform what might be appropriate, evidence is needed to quantify the outbreak risks associated with different restrictions and policies, so that they can be weighed against the social and economic cost in an objective way.

In this study we use an agent-based model, Covasim, to quantify the outbreak risks associated with a range of realistic restrictions. For an undetected infection being introduced to a setting with no community transmission, we aimed to estimate the risk of an outbreak and how this risk could be reduced with behavioural changes. The modelling is undertaken using parameters for the state of Victoria, Australia, based on a context of no community transmission.

## Methods

### Model overview

We used an established agent-based microsimulation model, Covasim [16, 17], developed by the Institute for Disease Modeling (USA) and previously adapted by the Burnet Institute to model the Victorian epidemic [18]. In brief, agents in the model are assigned an age (which affects their disease prognosis), a household, a school (for people age 5–18) or a workplace (for people aged 18–65), and can participate in a number of daily community activities including attending restaurants, pubs, places of worship, community sport, and small social gatherings. Super-spreading events are a key driver of COVID-19 transmission [19–22] and are accounted for in the model mechanistically in two ways. First, Covasim samples individual infectiousness from a negative binomial distribution such that some agents are more infectious and therefore have a higher probability of causing superspreading events [16, 18]. Second, the number of contacts an individual has in each setting is randomly sampled, with social contacts in particular being sampled from a negative binomial distribution, so some individuals are assigned many more contacts than others. This allows super-spreading events to occur in the model if a highly infectious individual also has many contacts. The model also includes testing, contact tracing and quarantine of close contacts, isolation of confirmed cases, masks, density limits in venues (e.g. 1 person per 4 square metres in restaurants), and other policy restrictions to prevent or reduce transmission in different settings (e.g. closing schools or venues).

Full details of the model structure and implementation, contact layers, transmission probabilities, and contact tracing capability are provided in Additional file 1. Code to run the simulations is available at https://doi.org/10.5281/zenodo.4784942.

### Testing, contact tracing and quarantine

Testing was modelled such that 50% of people with symptoms would seek testing, with a delay of 24 h between first symptoms and test-seeking. We assumed positive test results take 24 h to become available [23] and we assumed 75% compliance with self-quarantine while waiting for test results. Following a positive test, all household contacts in the model are notified by the confirmed case directly. Contact tracing for other contacts was assumed to take an additional 24 h (based on estimated average performance; see [24]), and we assumed it is able to identify 95% of people in workplaces, schools, childcare, and aged care; 50% in of people in venues such as restaurants, and 10% of people in community settings such as public transport. In the model, contact tracing for non-household contacts can be performed for a maximum of 250 newly diagnosed people per day, which is an estimate of Victoria’s tracing capacity during the second wave. Accounting for tracing capacity is important in large outbreaks, but we note that the analyses in this study focus on outbreaks that are much smaller than the tracing capacity, and thus our findings largely do not depend on this parameter. People identified as close contacts of a confirmed case are required to self-quarantine for 14 days, and we assume full compliance with this requirement. It was assumed that 90% of people with symptoms in quarantine would seek testing without delay. A full listing of tracing parameters by layer is provided in Additional file 1.

### Masks

A comprehensive meta-analysis [25] (conducted after two others [26, 27]), covering 41 studies of mask effectiveness concluded that that masks are associated with a reduction in infection for mask-wearers by a third compared to control groups. However, mask usage varies with settings, so we classified each contact layer in the model as having high, medium, or low usage. We therefore assumed masks would provide a reduction in transmission probability per contact of 30% in workplaces, entertainment venues, large events, and aged care; 25% in community settings, places of worship, public parks, social gatherings and on public transport; and 10% in cafes, restaurants, pubs and bars.

### Restriction levels

We defined a representative range of restriction levels from normal activity (Level 0) to the hard lockdown (Level 9), as shown in Table 1. These representative levels were based on the staged restrictions used in Victoria, but map approximately to restriction levels in many settings, such as the four-level alert system in New Zealand [28] or the three-tier system in the United Kingdom [29]. Full details of each policy and the effect on transmission in each contact layer are provided in Additional file 1.

**Table 1:**
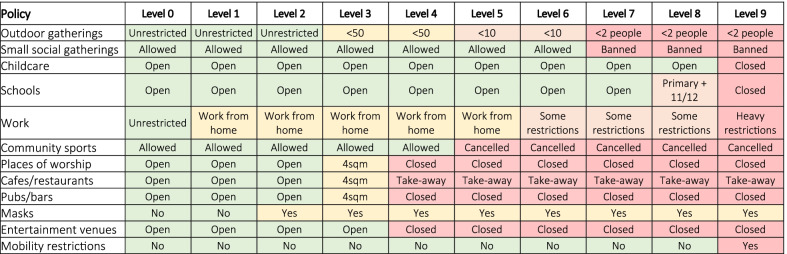
Combinations of policies included in each policy package examined. The specific effects of each policy on disease transmission is provided in Additional file 1

### Model calibration

The model was calibrated to the outbreak in Victoria over the June–August period [14], and the associated policy changes and interventions that were implemented over that period. Even though this analysis is based around the introduction of an infection in the context of no community cases, this calibration was used as a method for estimating and validating model parameters for the transmission probability per contact per day in a variety of settings, and the effectiveness of interventions.

### Outbreak analysis

We investigated the probability that a new infection would cause an outbreak if it were introduced to a community setting that had no existing infections. A 60-day period was modelled following the introduction of a single new infection under each restriction level, to represent the period at the start of an outbreak where containment would be managed by ongoing testing, tracing, and isolating procedures rather than being dominated by policy responses (e.g. wider lockdowns and closures prompted by the outbreak, which are uncertain and will vary from setting to setting).

When a new infection is introduced in the model, the epidemic trajectory is random and depends on factors such as who is initially infected (e.g., whether they have many workplace or community contacts, size of their household, or their individual transmissibility) and how quickly they are diagnosed. Therefore 1000 simulations were run for each restriction level, with different initial infections each time.

The are many possible ways to define and classify outbreaks based on metrics such as their size or growth rate. For the purposes of this analysis we have classified simulations as either *‘contained’* (the 7-day average of new cases per day was 0 after 60 days), *‘under control’* (the 7-day average of new cases per day was > 0 after 60 days, but did not exceed 5 during the simulation), or an *‘outbreak’* (the 7-day average of new cases per day reached > 5 within 60 days). These definitions were chosen based on whether a policy change or additional restrictions/intervention would likely be required. We have used the number of diagnoses rather than raw number of infections because this is a measurable quantity that could form the basis for potential policy responses. Individual daily new case counts are typically noisy, so policy decisions are often based on rolling average counts which reflect the trend of the outbreak. For example, the Victorian state government previously used the 14-day average as a guide for relaxing restrictions in late 2020. However, a 14-day window is likely too long to respond to a growing outbreak in a timely fashion, so we have used a 7-day average in this study. Australian state governments have implemented policy responses to small outbreaks, although risk tolerance varies from state to state. Western Australian and Queensland previously have imposed short lockdowns in response to single cases, whereas New South Wales had typically responded with lighter restrictions. We view 5 cases per day as representing a threshold that most states would be likely to act on. Finally, the 60-day simulation timeframe reflects the timescale of typical outbreaks, that grow or shrink approximately exponentially. The 60-day timeframe provides sufficient time for the outbreak to either be contained, or for it to grow much larger than 5 cases/day.

For each simulation it was recorded: (a) whether the infection was contained, under control, or led to an outbreak (as defined above); (b) days to first diagnosis; (c) cumulative number of infections at the time of first diagnosis; (d) days to reach a 7-day average of 5 cases per day; and (d) cumulative number of infections after 60 days.

### Sensitivity analysis: risk reduction strategies

To examine the scope for behavioural changes to complement restrictions, and the risks associated with complacency, we performed sensitivity analyses around the proportion of people with symptoms that seek testing, test-related delays, and test quarantine compliance, as shown in Table 2. Test related delays include delay to test and test turnaround time. The ‘delay to test’ is the time taken between a person becoming symptomatic, and seeking a test, if they get tested. The ‘test turnaround time’ is the time taken between the test being performed and the results becoming available. In Victoria, individuals must self-quarantine if they have been tested and are awaiting results. Thus, they do not quarantine during their delay to test, but do quarantine during the test turnaround time. The ‘test quarantine compliance’ is the proportion of people that comply with this requirement and quarantine until they receive their test results.

**Table 2:** Parameter scenarios examined for each restriction level

Scenario	Symptomatic test proportion	Delay to test (days)	Test turnaround time (days)	Test quarantine compliance
Baseline	0.5	1	1	0.75
Better				
Best case	0.75	1	1	1
More testing	0.75			
More test quarantine compliance				1
Worse				
Less test quarantine compliance				0.5
Slower test results			2	
Slower to seek testing		2		
Less testing	0.25			

## Results

### Outbreak probability

**Fig. 1:**
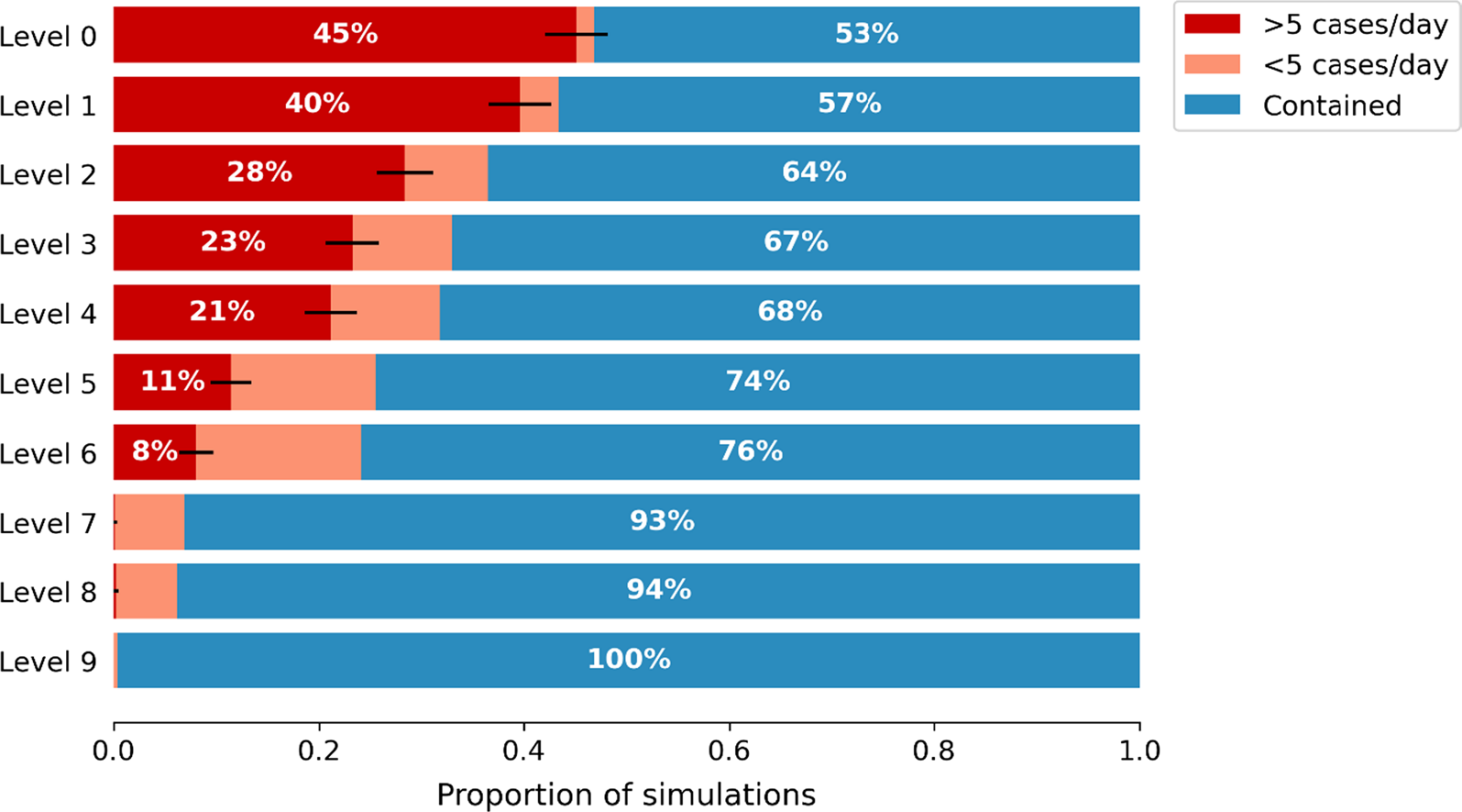
Outbreak probability. For each level of restrictions, the proportion of simulations where introducing an undiagnosed infection to a setting with zero transmission was contained (blue; defined as a 7-day average of 0 cases per day after 60 days), under control (orange; defines as a 7-day average of > 0 but < 5 diagnoses per day after 60 days), or led to an outbreak (red; defined as a 7-day average of > 5 cases per day after 60 days). The error bars show the 95% binomial confidence interval for the 1000 simulations performed, reflecting uncertainty in the estimation of the probability for the given number of model runs

For all restriction levels, a newly seeded undiagnosed case was contained in at least 50% of simulations (Fig. 1), but the chance of containment decreased if fewer restrictions were in place. As restrictions were eased, the likelihood of the situation not being contained but either “under-control” or an “outbreak” increased. With no restrictions, 45% of simulations resulted in an outbreak, compared to 40% if working from home was in place (Level 1), 28% with working from home + masks (Level 2), or 23% with working from home + masks + density limits on venues (Level 3).

**Fig. 2:**
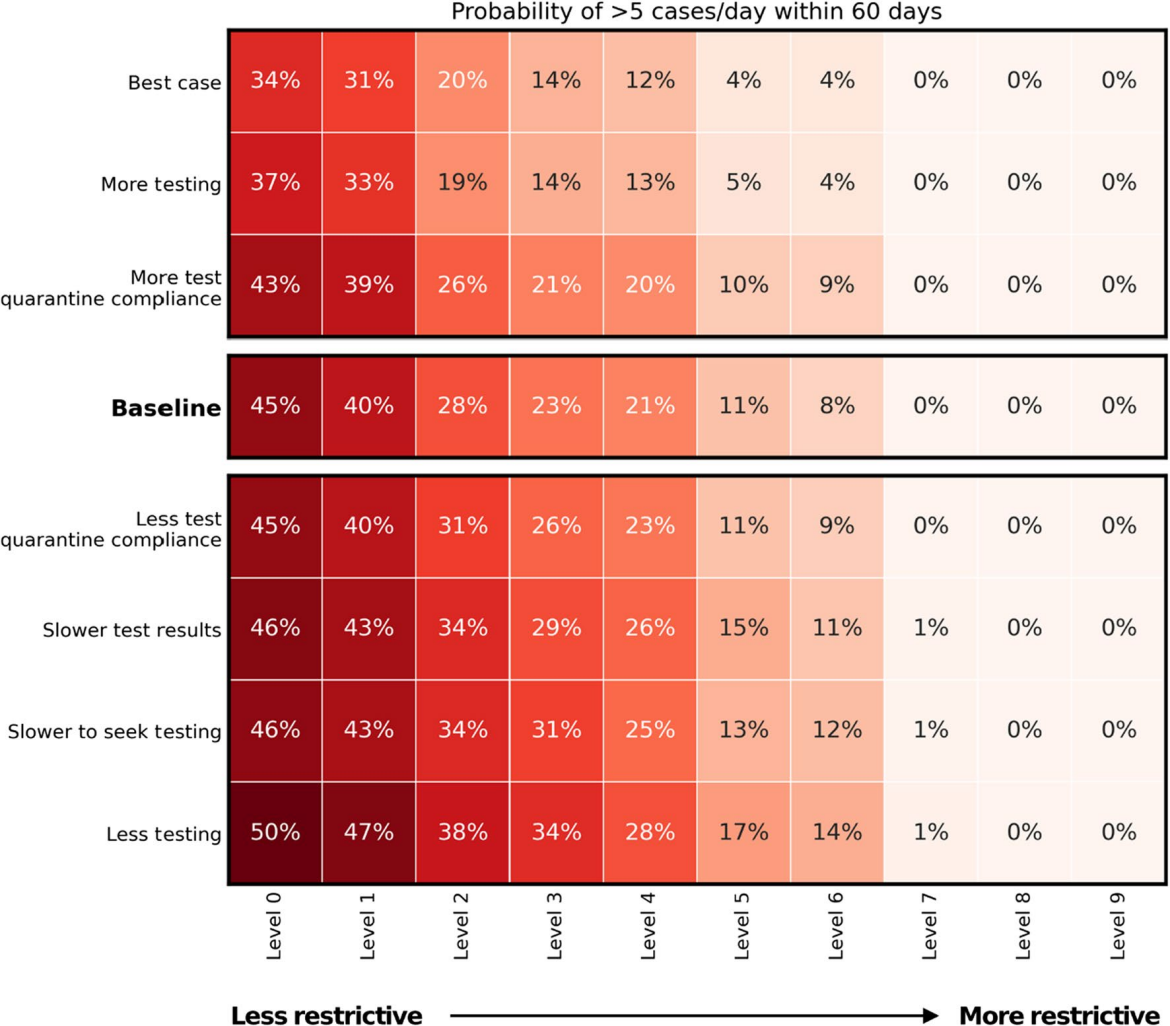
Sensitivity analysis for outbreak probability. Probability of the outbreak reaching > 5 cases/day within 60 days, for each restriction level and testing/compliance combination

In general, increasing the proportion of symptomatic people that seek testing is comparable or better than increasing restrictions by a single level (Fig. 2). For example, if a sustainable economic option were for businesses to operate with density limits (Level 3 restrictions) then the baseline probability of an outbreak is 23%. Increasing restrictions by closing pubs and restaurants entirely (Level 4 restrictions) would only decrease the risk by 2 percentage points. However, if the testing rate was increased such that 75% of symptomatic individuals sought testing, rather than 50%, then the risk decreases by 9 percentage points. Conversely, the ‘Less testing’ scenario in which only 25% of symptomatic people seek testing has an increase in risk of 11 percentage points, highlighting the large increase in risk associated with a drop in testing.

Changing the proportion of people that quarantine while waiting for tests results had a minimal effect on outbreak probability. At Level 3 restrictions, full compliance only decreased risk by 2 percentage points, while 50% compliance (rather than 75%) increased risk by 3 percentage points. This relatively small effect size is likely because we have assumed that test results are returned within 24 h, so the required quarantine period would be very small. The effect of quarantine compliance would likely be more pronounced if tests took longer to process.

### Outbreak time to first diagnosis

**Fig. 3:**
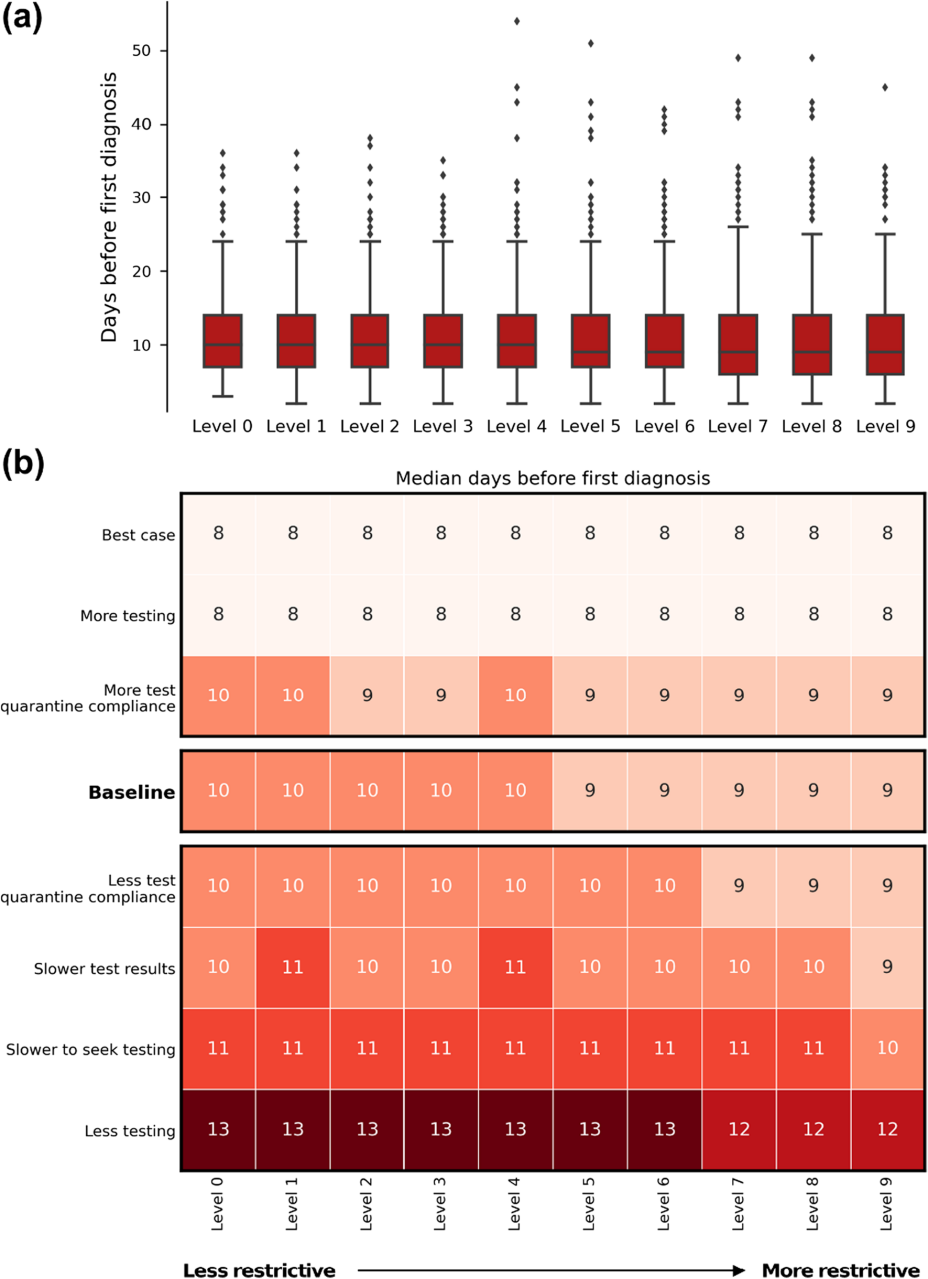
Time to first diagnosis. **a** For the baseline scenario, the distribution across the 1000 simulations sampled. **b** Median values for each restriction level and testing/compliance combination

The time taken to diagnose the first case reflects the incubation and pre-symptomatic period for COVID-19 and is mainly driven by the testing rate. Therefore, it remained fairly consistent across all of the levels of restrictions (Fig. 3). The median time to detect the outbreak was 10 days, which could decrease by up to 2 days with increased testing, or increase by up to 4 days if testing rates reduced.

### Outbreak size at first diagnosis

**Fig. 4:**
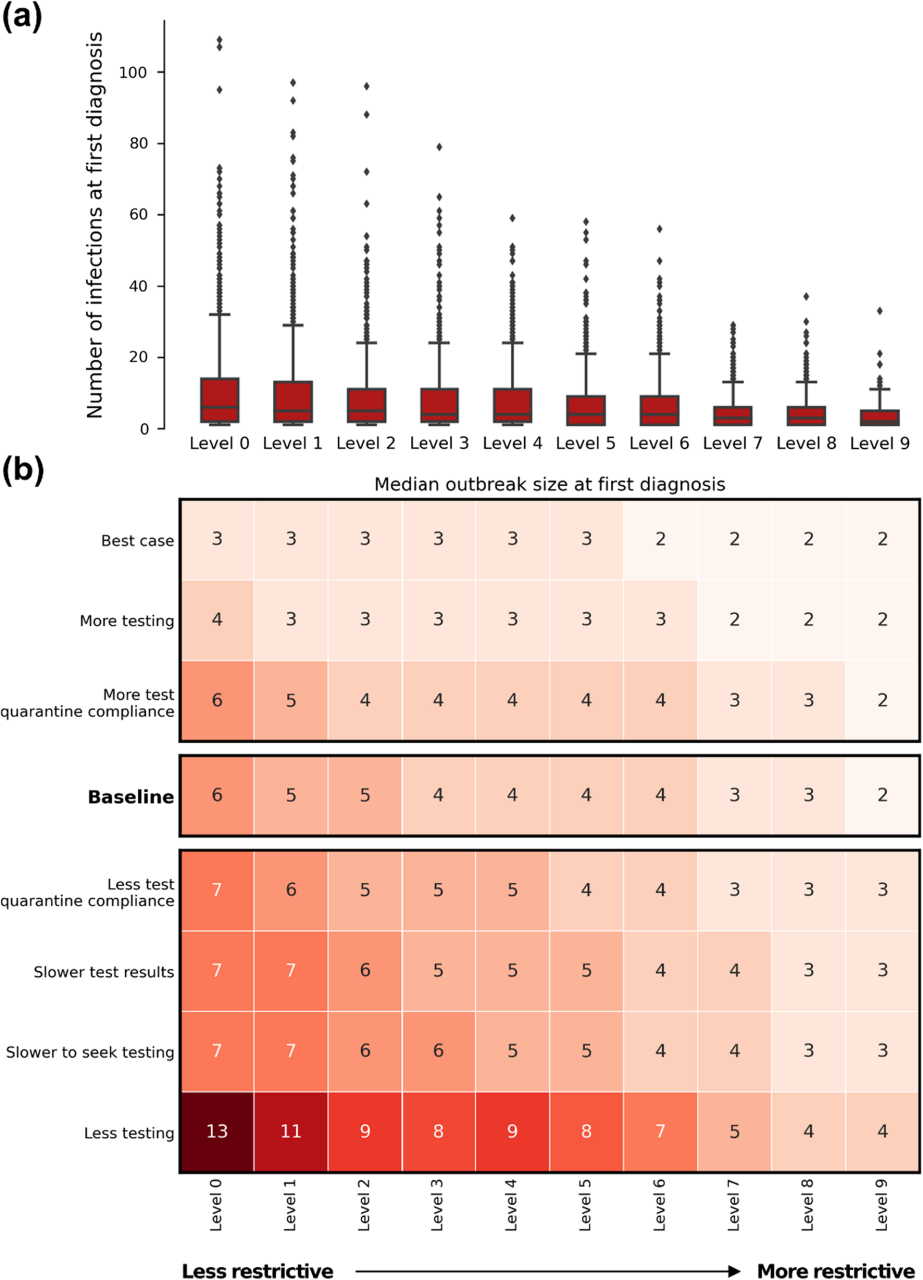
Outbreak size at first diagnosis. **a** For the baseline scenario, the distribution across the 1000 simulations sampled, **b** Median values for each restriction level and testing/compliance combination

When there were fewer restrictions and thus more rapid outbreak growth, the number of transmission events prior to the first diagnosis was higher (Fig. 4). With no restrictions and 50% of symptomatic people seeking testing, there were a median of 6 community infections by the time the first diagnosis was recorded. With less testing, this increases to a median of 13 cases when the first case is diagnosed.

### Outbreak time to 5 cases/day

**Fig. 5:**
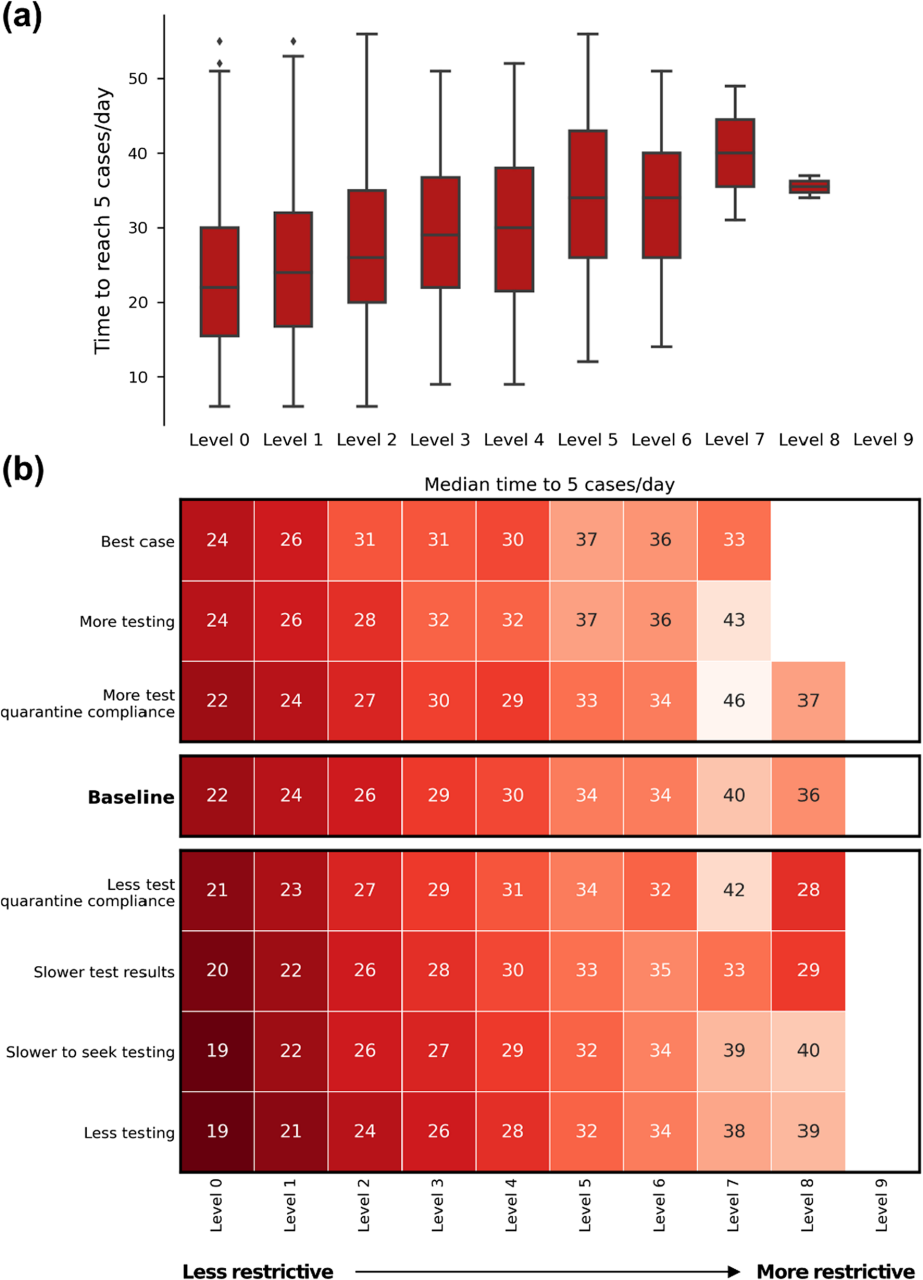
Time between the first case being diagnosed and reaching 5 cases/day. **a** For the baseline scenario, showing the distribution across the 1000 simulations sampled. **b** Median values for each restriction level and testing/compliance combination

If the outbreak was not contained or controlled, with no restrictions the outbreak took a median of 22 days from when the first case was diagnosed to when the outbreak reached a 7-day average of 5 diagnoses per day (Fig. 5). With greater restrictions in place, the growth rate was reduced. At high restriction levels, there were very few or no simulations that reached this threshold, so the distributions for Levels 8 and 9 mainly reflect the small number of samples rather than the growth rate of the outbreak. A larger number of samples could provide a more precise estimate of the growth rate, but we note that outbreaks under hard restrictions are associated with rare chains of transmission, and therefore the growth rate would primarily reflect these rare events rather than more general properties of transmission under those restrictions.

Overall, increases in symptomatic testing slightly increased the time to reach a 7-day average of 5 diagnoses per day, by around 2–3 days compared to baseline. This impact appears small compared to the change in outbreak risk associated with increased testing. However, increased testing means that more cases are diagnosed, which can increase the number of diagnosed cases per day even for the same total number of cases. Thus while increased testing is expected to decrease the growth rate of the epidemic due to more cases being quarantined and contact-traced, the increased number of diagnoses may partially mask this effect when looking at diagnosis-based metrics.

### Outbreak size after 60 days assuming no changes in behaviour or policy

**Fig. 6:**
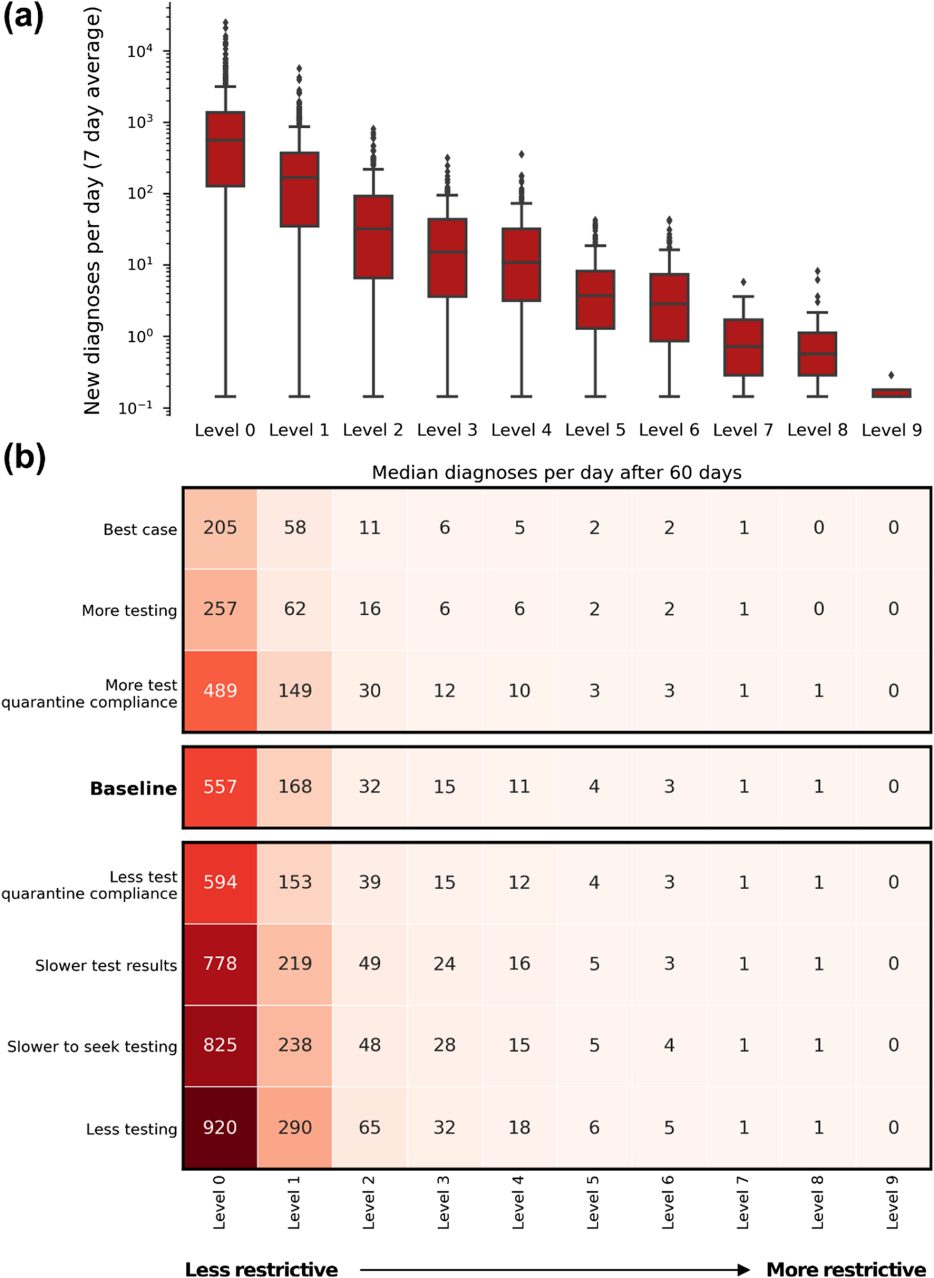
Number of new diagnoses/day (7 day average) after 60 days for each policy package, given that the outbreak was not contained (> 0 diagnoses/day after 60 days, 7 day average). **a** For the baseline scenario, the distribution across the 1000 simulations sampled. **b** Median values for each restriction level and testing/compliance combination

The size of the outbreak after 60 days exhibited considerable variation, highlighting the variability in outcome depending on the specifics of who is infected and how quickly they and their contacts are identified (Fig. 6). The distribution was also extremely long-tailed, with the largest possible outbreaks for each restriction level being much larger than the median or mean outcome (noting the logarithmic y-axis scale).

Even light restrictions (Level 1 and 2) resulted in much smaller outbreaks after 60 days, with median 7-day average of 32 diagnoses per day with working from home + masks (Level 2), compared to 557 diagnoses per day with no restrictions (Level 0).

We note that the simulations in Fig. 6 represent worst-case counterfactual scenarios where no policy interventions take place during the 60-day simulation window. In past Australian outbreaks, interventions such as reintroduction of restrictions and suburban testing blitzes have been implemented fairly quickly (with daily diagnoses cases < 30 cases/day). Similar responses would likely take place in future outbreaks, and these would change the trajectory of the outbreak compared to Fig. 6.

## Discussion

We used an agent-based model to investigate possible trajectories of a newly-seeded undiagnosed COVID-19 infection in the context of no community transmission in Victoria, Australia. Our results suggest that with no restrictions in place, there is a high probability that a single introduced case could trigger an outbreak. After a seed infection, it took a median 10 days (IQR = 7) to detect the first case, at which point there were estimated to be a median 6 cases (IQR = 12) already in the community. In 45% of simulations an outbreak with a 7-day average of > 5 diagnoses/day occurred. The results also suggest that light restrictions or behavioural changes could considerably reduce the risk of an outbreak. Working from home, working from home + masks, and working from home + masks + density limits on venues reduced the outbreak risk to 40%, 28% and 23% respectively.

These results suggest that relaxing restrictions even with no cases in the community may carry a high level of risk because a single incursion event could trigger an outbreak, and such incursion events are reasonably likely even with strict border controls. Travel-related incursions have already been observed multiple times, in Vietnam, New Zealand, and in the Australian states of Victoria, New South Wales, and South Australia. A number of studies have examined options for quarantine protocols and border controls [30–35]. A recent analysis suggests that under current Australian 14-day quarantine protocols for international arrivals and based on the prevalence of COVID-19 among arrivals to Australia, there is estimated to be approximately 2 to 6 infectious days in the community per 10,000 arrivals due to the imperfect test sensitivity and possibility of > 14 day incubation period of COVID-19 [34]. This means that any plans to increase international arrivals from high prevalence settings, even with a 14-day quarantine, must be considered in the context of local measures to maintain a constant level of outbreak risk within a setting. An integrated risk analysis is needed to inform debates on international travel, particularly regarding how to repatriate citizens or promote economic activity (e.g. international students).

Given the role of super-spreading events in SARS-CoV-2 transmission, relatively light policies aimed at reducing super-spreading events would be expected to greatly reduce transmission potential [36, 37]. We found that maintaining light restrictions even when there is no community transmission has ongoing benefits. For example, by maintaining a work-from-home policy and mandatory masks, the outbreak probability decreased to 28%, a decrease of 17 percentage points, with further decreases seen if bars and restaurants also had density limits. Moreover, maintaining light restrictions substantially reduced the growth rate of the epidemic if an outbreak did occur, providing more time to implement a response.

Both restrictions and testing contribute to outbreak containment—restrictions limit the number of opportunities for transmission by undiagnosed individuals, while testing enables isolation and contact tracing to effectively apply targeted restrictions on people at high risk of infection. However, due to the complex trade-off between testing, tracing, restrictions, and epidemic growth, the effect size of restrictions relative to increased testing is unknown. Using our model with a set of restriction levels based on Australian outbreak responses, we found that maintaining or increasing symptomatic testing was a highly effective way to reduce outbreak risk, often comparable with adding a level of restrictions. For example, promoting the importance of testing such that 75% of those with symptoms test resulted in a larger decrease in outbreak risk with Level 2 restrictions than closing bars and restaurants (Level 4 restrictions). Conversely if the symptomatic testing rate were to decrease to 25% then outbreak risk increased considerably, by up to 10 percentage points. These results highlight the importance of fast, widespread testing. Australian states have each responded to outbreaks in different ways, and it is notable that New South Wales has contained several outbreaks using an approach that leans on testing and tracing with only light restrictions to respond to outbreaks, whereas other states have maintained suppression with rapid short lockdowns, supporting our result that restrictions can be traded off against testing.

However, maintaining high levels of testing and timely testing (e.g. testing soon after the first symptoms) will be difficult in the context of limited or no community transmission. If several months have passed without a detected case, people are likely to assume that their symptoms are not caused by COVID-19 and may not test or delay testing to see if their symptoms are mild and/or naturally resolve. While it is true that in a setting with zero transmission, symptoms consistent with COVID-19 are unlikely to be caused by COVID-19 working to maintain high levels of symptomatic testing is critical. It increases the chance of detecting a new outbreak early, at a point when a small number of transmissions greatly influences the future trajectory of the outbreak.

Interestingly, there was only a small difference in outbreak risk depending on compliance with requirements to quarantine while waiting for test results. This is likely because in the scenarios we modelled, the number of infectious days in the community averted is limited because the test turnaround time is only one day. In the context of near-zero community transmission, the majority of people being tested are uninfected, and thus whether they quarantine or not does not make a difference to transmission. As a consequence, our results suggest it is worth exploring whether in a zero transmission setting there is a net benefit of removing the need to quarantine after testing, to increase symptomatic testing rates and detect new outbreaks more quickly. Then, if a new community case is detected, quarantine following testing could be reintroduced carte-blanche or for contacts of the case or within the relevant geographical area.

Our study builds on early work examining outbreak trajectories [35, 38–40], incorporating extensive data from the Victorian second wave that was not previously available, and including detailed contact layers, testing, and contact tracing. In March 2020, Hellewell et al. [38] estimated that with 20 initial cases and 60% of contacts being traced, less than 50% of outbreaks would be controlled. Huamaní [39] examined new outbreaks in Peru in April 2020, and estimated that there was an 80% chance that a single infection could be contained, but that this probability decreases quickly as the number of cases increases, with only a 20% chance of containment with 10 initial cases. Our findings based on more recent data are somewhat more pessimistic, with comparable outbreak probabilities even with just one initial infection, likely driven by improved estimates of the proportion of cases that are asymptomatic, as well as the more detailed transmission dynamics in our model.

While recent studies tend to focus on containment and management of large epidemics such as those in European or American settings, analyses of new outbreaks in settings with zero community transmission remain relevant for a significant number of countries that have achieved epidemic control. As vaccines against SARS-CoV-2 become available in 2021, understanding how outbreak risk changes depending on vaccine properties and coverage will be critical for informing border controls and outbreak response plans for these settings. Similarly, the outbreak risk associated with new variants could be different, if new variants differ in their infectiousness, incubation period, or their ability to escape prior immunity. Such changes could affect not only the downstream number of infections, but also the effectiveness of measures to contain the outbreak, such as contact tracing. The approaches used in this study can be readily extended to incorporate vaccines and new variants once data on their characteristics are available, and we plan to investigate a range of potential vaccine rollout scenarios and variant scenarios in future work.

## Limitations

The findings presented in this study are derived from an individual-based simulation model, Covasim. The model parameters are based on best-available data at the time of writing, including estimates of social mixing, contact networks, adherence to policies and quarantine advice, and disease characteristics (e.g. asymptomatic cases). There are several main limitations that impact the reported likelihood of an outbreak, including:

- It was assumed that after an extended period of low cases, 50% of symptomatic people would seek testing, which may be an overestimate (and hence the outbreak risk higher than estimated). However, we note that the symptomatic test rate here applies to people with COVID symptoms, and the testing rates for people with symptoms such as anosmia or fever is likely to be substantially higher than for people with mild respiratory symptoms only. Similarly, if the proportion of cases that are asymptomatic is different, the effectiveness of symptomatic testing in the community would also change accordingly.
- It was assumed that if a mandatory masks policy was in effect, compliance would be similar to the peak of the epidemic wave in August 2020 in Melbourne. However, it is likely that after an extended period of no community transmission, mask compliance would be somewhat lower. The overall effect of masks depends on both mask efficacy and compliance, and we note that our estimates for mask efficacy are conservative.
- An outbreak was defined as reaching a 7-day average of > 5 new diagnoses per day within 60 days. If a different threshold, time frame, or metric (e.g. infections rather than diagnoses) were used, then the probability reported would change, although this would not substantially impact the qualitative nature of the results.
- We presented some results indicating the likely outcome after 60 days under a worst-case assumption that there is no change in official policy, testing, or other individual behavior during this time. Given that it is unlikely that a change would not occur, the results in Fig. 6 should be interpreted as complementary to the results shown in Fig. 1 and indicative of the potential scale of the outbreak, rather than the projections for the actual likely scale.
- In this study, we have examined scenarios where a new infection is randomly seeded into the community—for example, an interstate traveller. If an incursion occurs within a sub-population subject to different testing policies (e.g., hotel quarantine staff that are regularly tested regardless of symptoms) then the overall level of risk is likely to be lower because the case is more likely to be diagnosed early on.

## Conclusions

In a zero-transmission setting, there is an ongoing risk of a case being introduced into the community, and this risk is likely to increase as the pandemic worsens elsewhere in the world. If life ‘returns to normal’ after a period of no community transmission, there is a considerable chance that even a single introduced case could trigger an outbreak. To stop an introduced case becoming an outbreak, it is critical to detect it as early as possible. Maintaining high testing rates remains a key factor in managing the risk of future outbreaks. Further, light restrictions can substantially increase the likelihood of it remaining contained or under-control. Testing and non-pharmaceutical interventions such as masks have a large benefit with minimal impact on broader well-being and the economy. However, community support may be difficult to maintain when there are no active cases, so ongoing government and community effort/engagement will be required to ensure that societies can be as open as possible, but at the same time detect and quickly contain introduced cases to manage the risk of needing greater restrictions.

## Supplementary Information

**Additional file 1.** Additional model details.

## Data Availability

Simulation code, analysis scripts, and saved output are publicly available and can be accessed at https://doi.org/10.5281/zenodo.4784942.

## Abbreviations

COVID-19: Coronavirus disease 2019
IQR: Interquartile range
NSW: New South Wales
SARS-CoV-2: Severe acute respiratory syndrome coronavirus-2
WHO: World Health Organization

## References

[1] Dong E, Du H, Gardner L (2020). An interactive web-based dashboard to track COVID-19 in real time. Lancet Infect Dis.

[2] Zhao S, Lin Q, Ran J, Musa SS, Yang G, Wang W (2020). Preliminary estimation of the basic reproduction number of novel coronavirus (2019-nCoV) in China, from 2019 to 2020: a data-driven analysis in the early phase of the outbreak. Int J Infect Dis.

[3] Musa SS, Zhao S, Wang MH, Habib AG, Mustapha UT, He D (2020). Estimation of exponential growth rate and basic reproduction number of the coronavirus disease 2019 (COVID-19) in Africa. Infect Dis Poverty..

[4] Mcaloon C, Collins Á, Hunt K, Barber A, Byrne AW, Butler F (2020). Incubation period of COVID-19: a rapid systematic review and meta-analysis of observational research. BMJ Open..

[5] Alene M, Yismaw L, Assemie MA, Ketema DB, Gietaneh W, Birhan TY (2021). Serial interval and incubation period of COVID-19: a systematic review and meta-analysis. BMC Infect Dis.

[6] Li Q, Guan X, Wu P, Wang X, Zhou L, Tong Y (2020). Early transmission dynamics in Wuhan, China, of novel coronavirus-infected pneumonia. N Engl J Med.

[7] Pollock AM, Lancaster J (2020). Asymptomatic transmission of covid-19. BMJ..

[8] Johansson MA, Quandelacy TM, Kada S, Prasad PV, Steele M, Brooks JT (2021). SARS-CoV-2 transmission from people without COVID-19 symptoms. JAMA Netw Open..

[9] Haas EJ, Angulo FJ, Mclaughlin JM, Anis E, Singer SR, Khan F (2021). Impact and effectiveness of mRNA BNT162b2 vaccine against SARS-CoV-2 infections and COVID-19 cases, hospitalisations, and deaths following a nationwide vaccination campaign in Israel: an observational study using national surveillance data. Lancet.

[10] Logunov DY, Dolzhikova IV, Shcheblyakov DV, Tukhvatulin AI, Zubkova OV, Dzharullaeva AS (2021). Safety and efficacy of an rAd26 and rAd5 vector-based heterologous prime-boost COVID-19 vaccine: an interim analysis of a randomised controlled phase 3 trial in Russia. Lancet.

[11] Voysey M, Clemens SAC, Madhi SA, Weckx LY, Folegatti PM, Aley PK (2021). Safety and efficacy of the ChAdOx1 nCoV-19 vaccine (AZD1222) against SARS-CoV-2: an interim analysis of four randomised controlled trials in Brazil, South Africa, and the UK. Lancet.

[12] Zhang Y, Zeng G, Pan H, Li C, Hu Y, Chu K (2021). Safety, tolerability, and immunogenicity of an inactivated SARS-CoV-2 vaccine in healthy adults aged 18–59 years: a randomised, double-blind, placebo-controlled, phase 1/2 clinical trial. Lancet Infect Dis.

[13] Wouters OJ, Shadlen KC, Salcher-Konrad M, Pollard AJ, Larson HJ, Teerawattananon Y (2021). Challenges in ensuring global access to COVID-19 vaccines: production, affordability, allocation, and deployment. Lancet.

[14] Victorian Department of Health and Human Services. Victorian coronavirus (COVID-19) data. 2020. https://www.dhhs.vic.gov.au/victorian-coronavirus-covid-19-data.

[15] New Zealand Ministry of Health. COVID-19: Source of cases 2020. https://www.health.govt.nz/our-work/diseases-and-conditions/covid-19-novel-coronavirus/covid-19-data-and-statistics/covid-19-source-cases.

[16] Kerr C, Stuart RM, Mistry D, Abeysuriya RG, Hart G, Rosenfeld K (2020). Covasim: an agent-based model of COVID-19 dynamics and interventions. medRxiv.

[17] Institute for Disease Modeling. Covasim model. https://github.com/InstituteforDiseaseModeling/covasim.

[18] Scott N, Palmer A, Delport D, Abeysuriya R, Stuart R, Kerr CC (2020). Modelling the impact of reducing control measures on the COVID-19 pandemic in a low transmission setting. Med J Aust..

[19] Adam DC, Wu P, Wong JY, Lau EHY, Tsang TK, Cauchemez S (2020). Clustering and superspreading potential of SARS-CoV-2 infections in Hong Kong. Nat Med.

[20] Endo A, Abbott S, Kucharski AJ, Funk S (2020). Estimating the overdispersion in COVID-19 transmission using outbreak sizes outside China. Wellcome Open Res.

[21] Althouse BM, Wenger EA, Miller JC, Scarpino SV, Allard A, Hébert-Dufresne L (2020). Superspreading events in the transmission dynamics of SARS-CoV-2: opportunities for interventions and control. PLOS Biol.

[22] Lau MSY, Grenfell B, Thomas M, Bryan M, Nelson K, Lopman B (2020). Characterizing superspreading events and age-specific infectiousness of SARS-CoV-2 transmission in Georgia, USA. Proc Natl Acad Sci.

[23] Australian Government Department of Health. Coronavirus (COVID-19) common operating picture. https://www.health.gov.au/resources/publications/coronavirus-covid-19-common-operating-picture.

[24] Abeysuriya R, Delport D, Hellard M, Scott N. Estimating risks associated with early reopening in Victoria. Policy brief. https://www.burnet.edu.au/projects/467_covasim_modelling_covid_19.

[25] IHME. COVID-19: what’s New for June 25, 2020. http://www.healthdata.org/sites/default/files/files/Projects/COVID/Estimation_update_062520.pdf.

[26] Liang M, Gao L, Cheng C, Zhou Q, Uy JP, Heiner K (2020). Efficacy of face mask in preventing respiratory virus transmission: a systematic review and meta-analysis. Travel Med Infect Dis.

[27] Chu DK, Akl EA, Duda S, Solo K, Yaacoub S, Schünemann HJ (2020). Physical distancing, face masks, and eye protection to prevent person-to-person transmission of SARS-CoV-2 and COVID-19: a systematic review and meta-analysis. Lancet..

[28] New Zealand Government. COVID-19 Alert System 2020 [Available from: https://covid19.govt.nz/alert-system/.

[29] UK Department of Health and Social Care. Local restriction tiers: what you need to know 2020. https://www.gov.uk/guidance/local-restriction-tiers-what-you-need-to-know.

[30] Clifford S, Quilty BJ, Russell TW, Liu Y, Chan Y-WD, Pearson CAB, et al. Strategies to reduce the risk of SARS-CoV-2 re-introduction from international travellers. 2020.

[31] Wells CR, Townsend JP, Pandey A, Moghadas SM, Krieger G, Singer B, et al. Optimal COVID-19 quarantine and testing strategies. 2020. 10.1038/s41467-020-20742-8 PMC7788536 33414470

[32] Ashcroft P, Lehtinen S, Angst DC, Low N, Bonhoeffer S. Quantifying the impact of quarantine duration on COVID-19 transmission. 2020. 10.7554/eLife.63704 PMC7963476 33543709

[33] Day M. Covid-19: eight day quarantine is as good as 14 for returning travellers, study finds. BMJ. 2020:m3047. 10.1136/bmj.m3047 32732429

[34] Sacks-Davis R, Cross W, Tidhar T, Palmer A, Heath K, Scott N, et al. traQ study: transparet risk assessment of quaranting. Final Report. https://burnet.edu.au/system/asset/file/4361/Final_Report_10November2020_Final.pdf. 2020.

[35] Adekunle A, Meehan M, Rojas-Alvarez D, Trauer J, Mcbryde E (2020). Delaying the COVID-19 epidemic in Australia: evaluating the effectiveness of international travel bans. Aust N Z J Public Health.

[36] Kain MP, Childs ML, Becker AD, Mordecai EA (2021). Chopping the tail: How preventing superspreading can help to maintain COVID-19 control. Epidemics..

[37] Frieden TR, Lee CT (2020). Identifying and interrupting superspreading events—implications for control of severe acute respiratory syndrome coronavirus 2. Emerg Infect Dis.

[38] Hellewell J, Abbott S, Gimma A, Bosse NI, Jarvis CI, Russell TW (2020). Feasibility of controlling COVID-19 outbreaks by isolation of cases and contacts. Lancet Glob Health.

[39] Huamaní C, Timaná-Ruiz R, Pinedo J, Pérez J, Vásquez L (2020). Condiciones estimadas para controlar la pandemia de COVID-19 en escenarios de pre y poscuarentena en el Perú. Rev Peru Med Exp Salud Publica.

[40] Tsou H-H, Cheng Y-C, Yuan H-Y, Hsu Y-T, Wu H-Y, Lee F-J (2020). The effect of preventing subclinical transmission on the containment of COVID-19: mathematical modeling and experience in Taiwan. Contemp Clin Trials..

