# Supplementary material for: Preventing a cluster from becoming a new wave in settings with zero community COVID-19 cases
Source: BMC Infect Dis. 2022 Mar 7;22:232. doi: 10.1186/s12879-022-07180-1 (PMC8899797; doi:10.1186/s12879-022-07180-1)
Supplement: Supplementary file 1 — Additional file 1. Additional model details. [file 12879_2022_7180_MOESM1_ESM.pdf]

Additional file 1

Additional model details

The agent-based model *Covasim* models the spread of COVID-19 by simulating a collection of agents representing people. Each agent in the model is characterised by a set of demographic and disease properties:

- Demographics:
  - Age (one-year brackets)
  - Household size, and uniquely identified household members
  - Uniquely identified school contacts (for people aged 5-18)
  - Uniquely identified work contacts (for people aged 18-65)
  - Average number of daily community contacts (multiple settings / contact networks modelled, described below)
- Disease properties:
  - Infection status (susceptible, exposed, recovered or dead)
  - Whether they are infectious (no, yes)
  - An over-dispersed individual transmission factor, sampled from a negative binomial distribution with unit mean and dispersion 0.45 based on Adam, Wu (1).
  - Whether they are symptomatic (no, mild, severe, critical; with probability of being symptomatic increasing with age, and the probability of symptoms being more severe increasing with age)
  - Diagnostic status (untested vs tested)

Transmission is modelled to occur when a susceptible individual is in contact with an infectious individual through one of their contact networks. The probability of transmission per contact is calibrated to match the epidemic dynamics observed and is weighted according to whether the infectious individual has symptoms, and the type of contact (e.g., household contacts are more likely to result in transmission than community contacts). Transmission dynamics depend on the structure of these contact networks, which are randomly generated but statistically resemble the specific setting being modelled. The layers included are described below, and the model parameters values are provided for each layer that was included.

Household contact network: household size and age structure

The household contact network was set up by explicitly modelling households. The households size distribution for Australia [5] was scaled to the number required for the number of agents in the simulation. Each person in the model was uniquely allocated to a household. To assign ages, a single person was selected from each household as an index, whose age was randomly sampled from the distribution of ages of the Household Reference Person Indicator in the 2016 Census for Greater Melbourne (2). The age of additional household members were then assigned according to Australian age-specific household contact estimates from Prem et al. (3), by drawing the age of the remaining members from a probability distribution based on the row corresponding to the age of the index member.

School and work contact networks

The school contact network was set up by explicitly modelling classrooms. Classroom sizes were drawn randomly from a Poisson distribution with mean 21 (4). People in the model aged 5-17 years were

assigned to classrooms with people their same age. Each classroom had one randomly selected adult (>21 years) assigned to it as a teacher. The result was that the school contact network was approximated as a collection of disjoint, completely connected clusters (i.e. classrooms).

Transmission in schools is influenced by age-specific disease susceptibility, and the age-specific probability of being symptomatic, which influences symptomatic testing interventions. In the model, people under 14 years have an odds ratio of 0.34 for acquiring infection relative to adults (5), and we use Victorian data to determine age-specific probability of being symptomatic, based on the percentage of positive contacts of confirmed cases who were symptomatic when they were tested. For this analysis it was additionally assumed that transmission risks in schools would be reduced by 50% relative to pre-COVID-19 based on the implementation of "COVID-Safe" plans following the second wave.

Similarly, a work contact network was created as a collection of disjoint, completely connected clusters of people aged 18-65. The mean size of each cluster was equal to the estimated average number of daily work contacts. Some workplaces are associated with a higher risk of infection, including healthcare settings, meat processing facilities, construction, warehousing and distribution, and are classified by the Department of Health and Human Services as high risk (6). In the model, we classified 15% of workplaces as high risk, based on labour force data from the Australian Bureau of Statistics (7). High risk workplaces were assigned a higher transmission probability, are less likely to be closed by restrictions (as many of these workplaces correspond to essential services.

Additional contact networks

An arbitrary number of additional networks can be added, but for this analysis we considered those most likely to be subject to policy change. Each network layer required inputs for: the proportion of the population who undertake these activities; the average number of contacts per day associated with these activities; the risk of transmission relative to a household contact (scaled to account for (in)frequency of some activities such as pubs/bars once per week); relevant age range; type of network structure (random, cluster [as per schools/workplaces]); and effectiveness of quarantine and contact tracing interventions.

Parameter values for each contact network

Table S1 shows the parameters that define each contact network in the model. Unless otherwise noted, parameters are derived in (8) from a mix of published and grey literature and a Delphi parameter estimation process. The columns of Table S1 refer to:

- **Mean contacts:** The average number of contacts per person in each network. Each person in the model has their individual number of contacts draw at random from a Poisson distribution with these values as the mean. For the social network layer, a negative binomial distribution was used with dispersion parameter 2 to account for a longer tail to the distribution.
- **Transmission probability:** The transmission probability per contact is expressed relative to household contacts, and reflects the risk of transmission depending on behaviour. For example, a casual contact in a public park is less likely to result in a transmission event compared to a contact on public transport.
- **Quarantine effect:** If a person is quarantined, the transmission probability is reduced by this factor. For example, an individual on quarantine at home would likely not work or use public transport, but they may still maintain their household contacts.

- **Population proportion:** Each network will only include a subset of the population e.g. every person has a household, but not every person regularly uses public transport.
- **Lower age/upper age:** Each network will only include agents whose age is within this range.
- **Clustered:** Here, we refer to a clustered network as one that consists of small groups people who are all connected to each other (e.g. classrooms), and where contacts do not change over time. This is compared to non-clustered networks, where contacts are randomly allocated. Non-clustered networks can either remain constant over time (e.g. social network) or have new contacts sampled each day (e.g. public transport).
- **Contact tracing probability –** the probability that each contact can be notified in order to quarantine

Table S1: Parameters for each of the networks in the model.

Layer	Mean contacts	Transmission probability (relative to households)	Quarantine effect	Population proportion	Lower age	Upper age	Clustered	^Contact tracing probability
Household	4	1	1	1	0	110	Y	1.00
Aged care	12	0.600	0.2	0.07	65	110	Y	0.95
Schools	21	0.124#	0.01	1	5	18	Y	0.95
Low risk work	5	0.282	0.1	1	18	65	Y	0.95
High risk work	5	0.847	0.1	1	18	65	Y	0.95
Church	20	0.043	0.01	0.11	0	110	Y	0.5
Community sport	30	0.071	0	0.34	4	30	Y	0.5
Childcare	20	0.274	0.01	0.545	1	6	Y	0.95
Community	1	0.100	0.2	1	0	110	N	0.1
Social	6	0.124	0.5	1	15	110	N	0.5
Entertainment	25	0.008	0	0.3	15	110	N	0.5
Cafes/Restaurants	8*	0.043	0	0.6	18	110	N	0.5
Pub/bar	8*	0.057	0	0.4	18	110	N	0.5
Transport	25	0.164	0.01	0.114	15	110	N	0.1
Public parks	10	0.028	0	0.6	0	110	N	0.1

^ Values are estimated or assumed by the authors. They do not represent data from, or the views of, the Victorian Department of Health and Human Services. The two estimates represent before and after assumed improvements in tracing systems, including the implementation of QR scanning systems in venues, media reports of locations of confirmed cases.
*Based on proposed indoor size limits of <10 in the roadmap

#Includes a 50% reduction from pre-COVID levels based on additional public health interventions

Testing and contact tracing

From 27th August onwards the Australian government has reported for each state the percentage of cases notifications within 24 hours of the test, and the percentage of close contacts notified within 48 hours of the positive test result (9). Recent estimates (17th September) suggest that in Victoria 100% of cases are notified in 24 hours of testing, and 99% of close contacts were notified within 48 hours of the positive test. In reality, the notification time and contact tracing time will be distributions (with these estimates suggesting that 24 hours and 48 hours are the tail ends, respectively), however the model is parametrised so that all tests and contact traces are completed at exactly the same time, and so single values are estimated as inputs. We therefore assumed that all tests are returned exactly 24 hours after they are taken, and all contacts take exactly 24 hours to be traced (the model uses daily

time steps so this was selected as more appropriate than the reported tail at 48 hours (9), or than assuming no delay).

Contact tracing was modelled by selecting individuals diagnosed each day, up to a maximum of 250 people each day representing an (unvalidated) estimate of contact tracing capacity in Victoria. For each person selected, their contacts were quarantined for 14 days with a network-specific probability of being detected (Table S1), reflecting differences in the level of difficult in identifying contacts in that network. The contact tracing capacity does not apply to household contacts, which are assumed to be directly notified by newly diagnosed individuals. The limited contact tracing capacity only affects outbreaks that have grown large enough to exceed the tracing capacity – this was the case during the Victorian second wave, but most of the results in this study concern small outbreaks that are well below the tracing capacity. Only the model calibration and results for low restriction levels in Figure 6 are expected to depend on the tracing capacity.

We also assumed 25% coverage of the *COVIDSafe* app with 24-hour tracing time.

<u>Model calibration</u>

The model was calibrated to the outbreak in Victoria over the June-September period, and the associated policy changes and interventions that were implemented over that period (Table S2).

	Pre-stage 3	Stage 3<br>Phased in from 2 July	Masks<br>23 July	Stage 4<br>5 August
Schools	Open	Restrictions		Closed
Workplaces	COVIDSafe plans	Restrictions		Heavier restrictions
Socialising	Size limits	Size limits		Curfew (ending on 28th Sep) and outdoor limits
Community sport	Going	Cancelled		
Pubs and bars	4 sq m rule	Closed		
Cafes and restaurants	4 sq m rule	Take-away only		
Places of worship	4 sq m rule	Closed		
Childcare	Open			Closed
Public parks	Open			Playgrounds closed
Public transport	Demand reduced indirectly			
Large events	Banned			
Entertainment venues	Closed			
Masks	No masks		Mandatory	

Table S2: Policy changes included in the model calibration process.

Testing was modelled by assigning a per day test probability to symptomatic and asymptomatic people that was fitted as part of calibration. We assumed some improvements over time, such that there were different inputs for testing and contact tracing for June-July and August-December, with the exact day the improvements occurred calibrated to fit the epidemic trajectory. We assumed that test results took 48 hours (exactly) to be processed initially and then 24 hours (exactly) after improvement.

Overall, the transmission probability per contact governs the rate of epidemic growth, and the testing parameters affect the daily diagnoses as well as the proportion of cases that go undiagnosed. We assume that the proportion of undiagnosed cases is reflected in the number of diagnoses relative to the number of hospitalizations, as severe cases are assumed to present at hospital

regardless of whether they have been tested or not. Thus, we used data on the number daily diagnoses and number of hospitalizations to enable simultaneous calibration of the testing and transmission parameters.

For the calibration shown in Figure S1, the model was initialised with a population of 100,000 agents. Due to uncertainties in the date and transmission dynamics of the original incursion events leading to the second wave, we initialized the simulation with 250 seed cases, corresponding to a point after the outbreak was already established. The overall transmission risk per contact (which multiplies the transmission probabilities in Table S1 for each layer) was varied such that when combined with inputs for the number of tests conducted over time and changes in contacts resulting from policy changes (e.g. community sports being cancelled and restaurants, cafes being take-away only when Stage 3 restrictions were introduced), the distribution of model outcomes was centred near the actual epidemic trajectory.

When calibrating, we fit the model transmission parameters under the assumption that the observed epidemic wave in June/July was the most likely outcome, which occurred in all simulations. In reality, it is possible that the second wave was an unlikely/unlucky outcome, or alternatively, that it could have been worse and was in fact a relatively lucky outcome, depending on the networks of seed cases and their contacts, as well as the overall transmission parameter. Therefore, we sampled over a set of initializations and transmission parameters, and only retained those runs where the seed/transmission parameter combination produced a projection that sufficiently matched the data – we considered the model to be a suitable fit if it was within 10% of the cumulative diagnosed cases each day. Figure S1 shows examples of the simulation runs used to estimate parameters for this study. To avoid overly penalizing mismatches in the initial stage of the outbreak, we start accumulating the cumulative case count after the 30 days, hence the model output in Figure S1(a) is offset accordingly. We note that the variability permitted in the cumulative case counts is dominated by how high the peak of the second wave is, and as the epidemic declines, the variability in new diagnoses per day by mid-September is somewhat smaller. Overall, approximately 700 of the 10000 proposed initializations were accepted. Many initializations were rejected because they diverged from the actual second wave early on, when case numbers are relatively low and the outcomes of each individual case therefore have a significant impact on the trajectory of the outbreak.

The distribution of transmission probability parameter values for the accepted initializations is shown in Figure S2.

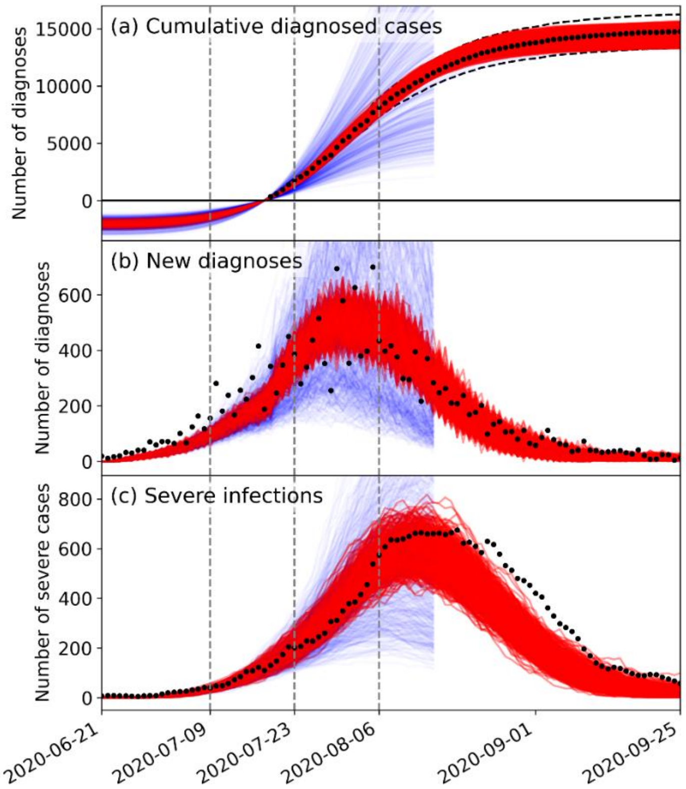

Figure S1: Model calibration to second wave in Victoria from June-September 2020. Vertical lines indicate when Stage 3 lockdowns took effect (9th July), masks were made mandatory (23rd July) and Stage 4 lockdowns took effect (6th Aug). Severe infections in the model represent infections requiring hospitalisation, and the corresponding data are for reported hospitalisations. Red lines indicate simulation runs that were accepted and used to obtain the baseline beta parameter distribution used in this study; blue lines show a representative sample of simulations that were rejected.

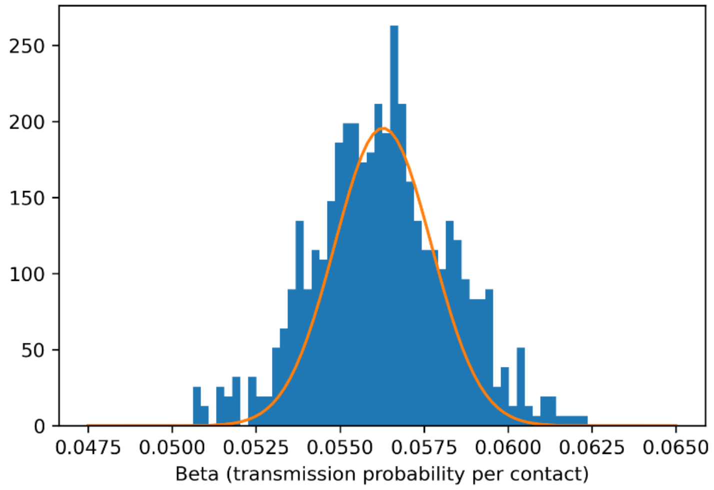

Figure S2: Distribution of baseline beta values for accepted calibration runs.

Disease prognosis

Table S3: Age-specific susceptibility, disease progression and mortality risks**.**

Age bracket	Relative susceptibility*	Prob[severe]#	Prob[critical] ##	Prob[death] ###
**0-5**	0.34	0.00004	0.0004	0.00002
**6-12**	0.34	0.00004	0.0004	0.00002
**13-15**	0.34	0.0004	0.00011	0.00006
**16-19**	1	0.0004	0.00011	0.00006
**20-29**	1	0.011	0.0005	0.0003
**30-39**	1	0.034	0.00123	0.0008
**40-49**	1	0.043	0.00214	0.0015
**50-59**	1	0.082	0.008	0.006
**60-69**	1	0.118	0.0275	0.022
**70-79**	1.24	0.166	0.06	0.051
**80+**	1.47	0.184	0.10333	0.093

*Zhang et al. (5) found children <14 had 34% less susceptibility to adults, and people>65 years had 47% increased susceptibility

(10, 11); ## (11); ### (10-12)

Policies

The effect of each policy is detailed below summarized from (8), showing the impact on the transmission probability per contact, and/or the number of contacts in the network. Policies that reduce the number of contacts in the network better preserve the clustering associated – for example, the 'Work from home' policy reduces the number of workplace contacts to model the same people working from home every day.

Large events cancelled
- Large event transmission reduced by 100%

Entertainment venues closed
- Entertainment transmission reduced by 100%

Cafes/restaurants open with 4sqm physical distancing
- Cafes/restaurants transmission reduced by 50%

Pubs/bars open with 4sqm physical distancing
- Pubs/bars transmission reduced by 50%

Churches/places of worship open with 4sqm physical distancing
- Church/places of worship transmission reduced by 60%

Work from home where possible
- Household transmission increased by 10%
- Work transmission reduced by 36%
- Additional community transmission reduced by 33%
- Public transport transmission reduced by 33%

Outdoor gatherings limited to <10 people
- Additional community transmission reduced by 20%
- Entertainment transmission reduced by 100%
- Public transport transmission reduced by 50%
- Public parks transmission reduced by 40%

Stage 3, Melbourne and Mitchell Shire, additional impacts
- Household transmission increased by 10%
- School transmission decreases by 85%
- Community sport transmission reduced by 85%
- Cafes/restaurants transmission reduced by 85%

Community sports cancelled
- Community sport transmission reduced by 100%

Cafes/restaurants takeaway only
- Cafes/restaurants transmission reduced by 100%

Pubs/bars takeaway closed
- Pubs/bars transmission reduced by 100%

Churches and places of worship closed
- Church/places of worship transmission reduced by 100%

Aged care improvements
- Aged care transmission reduced by 50%

Mandatory masks
- Work transmission reduced by 30%
- Additional community transmission reduced by 25%
- Church/places of worship transmission reduced by 25%
- Entertainment transmission reduced by 30%
- Cafes/restaurants transmission reduced by 10%
- Pubs/bars transmission reduced by 10%
- Public transport transmission reduced by 30%
- Public parks transmission reduced by 25%
- Large event transmission reduced by 30%
- Social gatherings transmission reduced by 25%
- Aged care transmission reduced by 30%
- Schools: 0% (assumed not mandatory in these projections)

Small social gatherings banned
- Stage 3: social contact transmission reduced by 67%
- Stage 4: social contact transmission reduced by 90%

Childcare closed
- Childcare transmission reduced by 100%

Schools closed
- School transmission reduced by 100%

Mobility restrictions
- Public transport transmission reduced by 80%
- General community transmission reduced by 70%

Stage 4 work restrictions
- Low risk work transmission reduced by 90%
- High risk work transmission reduced by 40%

50% reduction in transmissibility in Schools
- School transmission reduced by 50%

Outdoor gatherings limited to 50 people
- Public transport transmission reduced by 20%

References

1. Adam DC, Wu P, Wong JY, Lau EHY, Tsang TK, Cauchemez S, et al. Clustering and superspreading potential of SARS-CoV-2 infections in Hong Kong. Nature Medicine. 2020;26(11):1714-9.
2. Australian Bureau of Statistics (ABS). Australian Bureau of Statistics 2016, Census of Population and Housing, TableBuilder. Findings based on use of ABS TableBuilder data. https://www.abs.gov.au/ausstats/abs@.nsf/web+pages/Citing+ABS+Sources#TableBuilder i. 2020.
3. Prem K, Cook AR, Jit M. Projecting social contact matrices in 152 countries using contact surveys and demographic data. PLoS computational biology. 2017;13(9):e1005697.
4. https://www.study.vic.gov.au/en/study-in-victoria/victoria’s-school-system/Pages/default.aspx.
5. Zhang J, Litvinova M, Liang Y, Wang Y, Wang W, Zhao S, et al. Changes in contact patterns shape the dynamics of the COVID-19 outbreak in China. Science. 2020;368(6498):1481-6.
6. Victorian Department of Health and Human Services. High risk workplaces. https://www.dhhs.vic.gov.au/workplace-obligations-covid-19#how-do-i-know-if-my-businesses-is-a-high-risk-workplace.
7. Australian Bureau of Statistics (ABS). Census data; labour force. https://guest.censusdata.abs.gov.au/webapi/jsf/tableView/tableView.xhtml. 2016.
8. Scott N, Palmer A, Delport D, Abeysuriya R, Stuart R, Kerr CC, et al. Modelling the impact of reducing control measures on the COVID-19 pandemic in a low transmission setting. . Med J Aust. 2020;Accepted for publication. https://www.mja.com.au/journal/2020/modelling-impact-reducing-control-measures-covid-19-pandemic-low-transmission-setting.
9. Australian Government Department of Health. Coronavirus (COVID-19) common operating picture. https://www.health.gov.au/resources/publications/coronavirus-covid-19-common-operating-picture.
10. Verity R, Okell LC, Dorigatti I, Winskill P, Whittaker C, Imai N, et al. Estimates of the severity of coronavirus disease 2019: a model-based analysis. The Lancet Infectious Diseases. 2020;20(6):669-77.
11. Centers for Disease Control and Prevention COVID-19 Response Team. Severe outcomes among patients with coronavirus disease 2019 (COVID-19)—United States, February 12-March 16, 2020. MMWR Morb Mortal Wkly Rep. 2020;69(12):343-6.
12. Ferguson N, Laydon D, Nedjati Gilani G, Imai N, Ainslie K, Baguelin M, et al. Report 9: Impact of non-pharmaceutical interventions (NPIs) to reduce COVID19 mortality and healthcare demand. 2020.
